# A randomised translational trial of lifestyle intervention using a 3-tier shared care approach on pregnancy outcomes in Chinese women with gestational diabetes mellitus but without diabetes

**DOI:** 10.1186/s12967-014-0290-2

**Published:** 2014-10-28

**Authors:** Xilin Yang, Huiguang Tian, Fuxia Zhang, Cuiping Zhang, Yi Li, Junhong Leng, Leishen Wang, Gongsu Liu, Ling Dong, Zhijie Yu, Gang Hu, Juliana CN Chan

**Affiliations:** Department of Epidemiology and Biostatistics, School of Public Health, Tianjin Medical University, Tianjin, 300070 China; Department of Medicine and Therapeutics, Hong Kong Institute of Diabetes and Obesity, The Chinese University of Hong Kong and The Chinese University of Hong Kong-Prince of Wales Hospital-International Diabetes Federation Centre of Education, Hong Kong, China; Tianjin Women and Children’s Health Centre, Tianjin, China; Population Cancer Research Program and Department of Pediatrics, Dalhousie University, Halifax, Canada; Chronic Disease Epidemiology Laboratory, Pennington Biomedical Research Center, Baton Rouge, Louisiana USA

**Keywords:** Randomised controlled trial, Gestational diabetes mellitus, Shared care, Self-monitoring of blood glucose, Lifestyle intervention, Macrosomia, Large for gestational age

## Abstract

**Background:**

There are no randomised controlled trials to demonstrate whether lifestyle modifications can improve pregnancy outcomes of gestational diabetes mellitus (GDM) diagnosed by the International Association of Diabetes and Pregnancy Study Group’s (IADPSG) criteria. We tested the effectiveness of lifestyle modifications implemented in a 3-tier’s shared care (SC) on pregnancy outcomes of GDM.

**Methods:**

Between December 2010 and October 2012, we randomly assigned 700 women with IADPSG-defined GDM but without diabetes at 26.3 (interquartile range: 25.4-27.3) gestational weeks in Tianjin, China, to receive SC or usual care (UC). The SC group received individual consultations and group sessions and performed regular self-monitoring of blood glucose compared to one hospital-based education session in the UC group. The outcomes were macrosomia defined as birth weight ≥ 4.0 kg and the pregnancy-induced hypertension (PIH).

**Results:**

Women in the SC (n = 339) and UC (n = 361) groups delivered their infants at similar gestational weeks. Birth weight of infants in the SC group was lower than that in the UC group (3469 vs. 3371 grams, P = 0.021). The rate of macrosomia was 11.2% (38/339) in the SC group compared to 17.5% (63/361) in the UC group with relative risk (RR) of 0.64 (95% CI: 0.44-0.93). The rate of PIH was 8.0% (27/339) in the SC compared to 4.4% (16/361) in the UC with RR of 1.80 (0.99-3.28). Apgar score at 1 min < 7 was lower but preeclampsia was higher in the SC than in the UC.

**Conclusions:**

Lifestyle modifications using a SC system improved pregnancy outcomes in Chinese women with GDM.

**Trial registration:**

Clinicaltrials.gov; NCT01565564.

**Electronic supplementary material:**

The online version of this article (doi:10.1186/s12967-014-0290-2) contains supplementary material, which is available to authorized users.

## Background

Gestational diabetes mellitus (GDM) increases the risk of adverse pregnancy outcomes including macrosomia [1], preterm birth [1,2], shoulder dystocia, birth trauma and neonatal morbidities [3-5]. GDM also predisposes the offspring to high risk of childhood obesity [6], and the mothers to high risk of diabetes in the long run [7]. In the Hyperglycaemia and Adverse Pregnancy Outcomes (HAPO) Survey [8], there were strong and continuous associations of maternal plasma glucose levels with increased birth weight and C-peptide levels in cord-blood with no apparent thresholds. Two randomised controlled clinical trials evaluated the efficacy of treatment of mild GDM on pregnancy outcomes [9,10]. The Australian Carbohydrate Intolerance Study in Pregnant Women (ACHOIS) [10] reported that intensive intervention in women with GDM defined by a 75-gram 2-hour oral glucose tolerance test (OGTT) and the World Health Organization’s criteria [11] reduced the rate of serious perinatal complications (defined as death, shoulder dystocia, bone fracture, and nerve palsy). Among individual pregnancy outcomes, the rates of macrosomia and preeclampsia were significantly reduced [10]. Another multicenter randomised trial from the US [9] reported that intensive intervention in women with GDM defined by a 100-gram 3-hour (OGTT) and the Fourth International Workshop-Conference on GDM’s criteria [12] did not reduce the predefined composite endpoint of neonatal morbidity and its components (stillbirth or neonatal death, hypoglycemia, etc.) but significantly reduced the rates of macrosomia, shoulder dystocia and pregnancy-induced hypertension (PIH) [9]. In 2010, the International Association of Diabetes and Pregnancy Study Groups (IADPSG) recommended new criteria to define GDM with a much lower cutoff point for the fasting glucose than previous criteria for diagnosis of GDM [13], which diagnosed more cases in Asian and European pregnant women [14,15]. However, there is no randomised trials to demonstrate that intensive intervention is able to achieve similar effects on pregnancy outcomes as among GDM diagnosed by either the WHO’s criteria [11] or the Fourth International Workshop-Conference on GDM’s criteria [12].

In 1998, Tianjin implemented a universal screening for GDM [16]. Between 1999 and 2008, the prevalence of GDM in Tianjin had increased from 2.4% to 6.8% [17]. Although diabetes education has been shown to be effective in improving glycaemic control [18], the challenge lies in care organization to maximise the synergism between primary, secondary and tertiary care [19]. This study tested that lifestyle modification implemented in a shared care (SC) system among care providers was able to reduce adverse pregnancy outcomes as compared to usual care among women with GDM diagnosed by the IADPSG’s criteria [13]; and evaluated the feasibility of utilizing the 3-tier care delivery to provide a pragmatic solution to the increased need for medical care for these GDM women.

## Methods

### Study population

Tianjin is a metropolitan city in Northern China, ranking fourth in population size (12.8 millions) among Chinese cities. In urban Tianjin, antenatal care was shared by a 3-tier prenatal care system consisting of 1). 65 primary hospitals; 2). 6 district-level Women and Children’s Health Centres (WCHC) and other secondary obstetric hospitals; and 3). A city-level Tianjin WCHC (TWCHC) and other tertiary hospitals. Antenatal care was delivered in a relatively structured manner [1,16]. All pregnant women were registered at a primary hospital with regular antenatal clinic visits until 32^nd^ gestational week. Then they would be referred to one of the 25 secondary or tertiary obstetric hospitals at their choice where they would be managed till delivery. All pregnant women were offered a non-fasting 50-gram 1-hour glucose challenge test (GCT) at primary hospitals between 24^th^ and 28^th^ weeks of gestation. Women with plasma glucose (PG) at GCT ≥7.8 mmol/L were referred to TWCHC GDM Clinic, where they underwent a standard 75-gram 2-hour oral glucose tolerance test (OGTT). GDM was diagnosed according to the IADPSG’s criteria, i.e., having met any of the cutoff points: fasting PG ≥5.1 mmol/L, 1-hour PG ≥10.0 mmol/L or 2-hour PG ≥8.5 mmol/L [13].

### Study design

**Figure 1:**
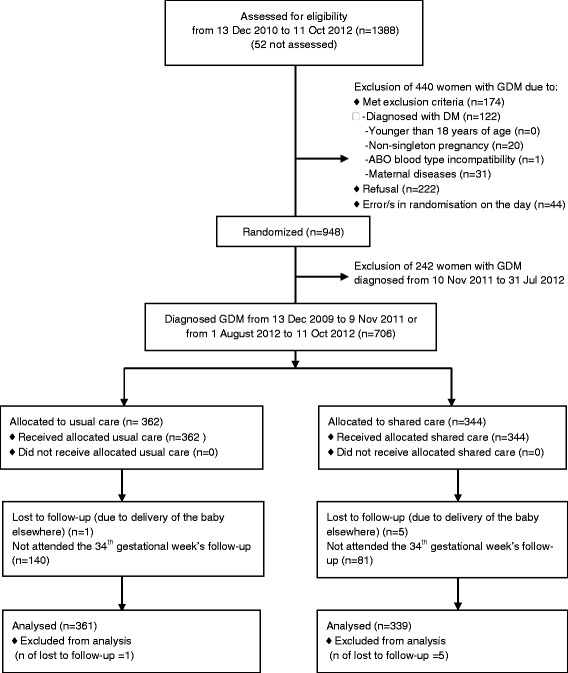
**Patient flow diagram.**

This was a randomised controlled effectiveness trial nested in the 3-tier prenatal care system of Tianjin, China. All women confirmed with GDM were invited to participate in the trial unless they had one or more of the following exclusion criteria: 1). The OGTT had met the diagnostic criteria of diabetes, i.e., fasting PG ≥ 7.0 mmol/L, 2-hour PG ≥ 11.1 mmol/L or HbA_1c_ ≥ 6.5% (48 mmol/mol) [20]; 2). Younger than 18 years of age; 3). Non-singleton pregnancy; 4). Maternal-foetal ABO blood type incompatibility; and 5). Maternal diseases such as chronic hypertension, thyrotoxicosis, prepregnancy diabetes and use of long-term medications that might affect glucose metabolism. Between 13 December 2010 and 11 October 2012, 19847 pregnant women were screened at primary hospitals and 2921 women were referred to TWCHC who underwent the 75-gram OGTT. Of them,1440 women were diagnosed to have GDM and we assessed 1388 women with GDM for their eligibility for the study (52 women with GDM diagnosed on Saturday were not invited to participate in the late stage of the fieldwork due to manpower limitations). Of them, 440 women with GDM were excluded due to having met one or more of exclusion criteria, refusal to participate, there being error/s in randomisation on the day (Figure 1). A total of 948 women were randomly assigned to receive either shared care (SC) or usual care (UC). However, during Nov 2010 to July 2011, separate areas for intervention/follow-up in the two groups were unavailable due to renovation of the whole building of TWCHC and data collection from the UC women was performed by the intervention staff members, the 242 women entering the trial during this period also received unintentional intervention. Therefore these women were excluded from the analysis. Among the 706 women entering the trial not during that period, one woman in the UC group and five women in the SC group delivered their infant outside Tianjin, 700 women (SC = 339; UC = 361) were available in the analysis (Figure 1). A simple randomization procedure without replacement (i.e., by the time sequence of visits to the clinic and a list of priori computer-generated random assignment status by X.Y.) was used to perform the random assignment by the intervention team members. The women with GDM in the trial but not the research team members were masked to the random assignment.

### Usual care

As usual practice, all women with GDM were offered a group education class lasting for 30–40 minutes at the TWCHC GDM clinic delivered by a diabetes educator who was not part of the SC team. During this session, they received advice on diet and physical activity but were not specifically taught to perform self monitoring of blood glucose (SMBG). For the UC women, insulin treatment was recommended if HbA_1c_ ≥6.5% (48 mmol/mol**)** during follow-up. However, use of insulin after being hospitalised for delivery was not standardised. The UC and SC care protocol is listed in Additional file 1.

### Shared care

We adapted the protocol of the intervention arm in the ACHOIS trial [10] to suit the cultural needs in our study for women assigned to the SC group. The additional intervention of SC was delivered by an intervention team from TWCHC, consisting of trained nurses and doctors. All women in the SC group were offered an individualised dietary advice and physical activity counselling at entry to the trial. Different energy intakes were recommended based on prepregnacy body mass index (BMI) classification for Chinese adults [21] with obese/overweight women being advised to have moderate restriction in total energy, i.e., 35 kcal/day/kg for one kilogram body weight per day in pregnancy for women with pre-pregnancy BMI at <18.5 kg/m^2^; 30–35 kcal/day for one kilogram body weight in pregnancy for women with pre-pregnancy BMI at 18.5-23.9 kg/m^2^; 25–30 kcal/day/kg for women with pre-pregnancy BMI at 24.0-27.9 kg/m^2^; 25 kcal/day/kg for women with pre-pregnancy BMI at 28.0 kg/m^2^ and more. All women were asked to be engaged in at least 30 minutes of light to moderate physical activity daily, i.e., walking. All women were offered a free glucose meter with memory function and free test strips. They were asked to perform SMBG, 4 times a day for the initial two weeks and then daily at different time points in rotation (pre-breakfast and 2 hours after three meals). The target of glycaemic control was ≥3.5- ≤ 5.1 mmol/l for fasting capillary blood glucose and ≤7.0 mmol/L for 2-hour postprandial capillary blood glucose up to 36^th^ gestational week and ≤8.0 mmol/L from 36^th^ week onwards. If blood glucose values exceeded the target values two times or more during a 2-week interval or the 2-hour postprandial capillary blood glucose exceeded 9.0 mmol/L once during a 1-week period, insulin therapy would be recommended by the intervention team and started by a senior obstetrician. At 30^th^ and 34^th^ gestational weeks, women in the SC group were offered two additional individualised sessions to reinforce diet, physical activity and SMBG. In addition to individualised counselling at TWCHC, women in the SC group were offered group education sessions each lasting for 2 hours at 27^th^, 29^th^ and 33^rd^ gestational weeks at TWCHC or a place close to their neighbourhood contingent upon their gestational week at diagnosis of GDM. The obstetric care of the SC women was the same as the UC women.

### Primary and secondary clinical outcomes

The primary outcome was macrosomia defined as birth weight ≥4000 grams. We also used large for gestational age (LGA) as a clinical outcome defined using the Tianjin local reference. Standard birth weights were obtained from all hospital deliveries for calibration to estimate LGA. The hospital discharge notes and the electronic medical records were retrieved from the Central Antenatal Care Database of Tianjin at the end of the study. The secondary outcome was incident PIH. The hospital discharge notes and the electronic medical notes for the entire antenatal care process were retrieved from the Central Antenatal Care Database of Tianjin at the end of the study. The case notes of PIH diagnosis and episodes of high BP were reviewed by an obstetrician (L.D.) who was not involved in the management of the women of GDM and masked to the assignment status. Incident PIH was diagnosed by first presence of gestational hypertension or preeclampsia/eclampsia after entering the trial. Gestational hypertension was defined as systolic/diastolic blood pressure (SBP/DBP) ≥ 140/90 mmHg; preeclampsia was defined as SBP/DBP ≥ 140/90 mmHg with proteinuria (+or more). Priori set powers were 85% to detect a 40% risk reduction for macrosomia and 80% to detect a 37% risk reduction for PIH at a 5% type I error if two-sided likelihood ratio test was used, which seemed to be achievable [9].

### Data collection

Before the fieldwork, fieldwork manuals were compiled and all personnel involved in the trial received at least 1-day training on protocols, intervention, care processes. The intervention team received at least 1-week additional training on intervention techniques. A series of pilot studies were conducted to confirm the feasibility in administering the questionnaires and streamline data collection. Data were collected in a longitudinal way starting from the first antenatal visit and screening visit for GDM at primary hospitals, and visits at the TWCHC GDM clinic and at postpartum. The patient health questionnaire (PHQ)-9 was a validated tool to detect depression in general and diabetic Chinese populations in Hong Kong [22-24] which was also administered at 34^th^ gestational week. Physical activity was collected with a validated questionnaire in 2002 China National Nutrition and Health Survey [25] at 34^th^ gestational week. A 24-hour food recall method was used to estimate energy intake. Maternal body weight and other maternal and neonatal outcome measures were documented at routine antenatal visits and delivery.

Ethical approval was obtained from the Clinical Ethics Committee of TWCHC and informed consent was obtained from all participants.

### Statistical analysis

The Statistical Analysis System (Release 9.30; SAS Institute, Cary, NC) was used to analyse the data. All data were expressed as mean (standard deviation, SD) or median (interquartile range, IQR) as appropriate. The intention-to-treat principle was followed in the data analysis. Body weight at the first antenatal care visit was treated as prepregnancy body weight due to relatively stable maternal weight in the first trimester of pregnancy [26]. Preterm birth was defined as gestational age at term less than 37 weeks. We used Chi-squared test (or Fisher’s exact test where appropriate) to compare categorical variables and the Student’s t test for continuous variables. We used Wilcoxon two-sample test if normal distribution was rejected by checking the Q-Q plot. The effects of SC compared to UC on predefined and post-hoc pregnancy outcomes were expressed as relative risk (RR) and relative risk reduction and their 95% confidence intervals (CI). Relative risk was estimated by prevalence ratio [27].

Subgroup analysis was performed to examine the effect size of SC among women with GDM diagnosed by the IADPSG’s criteria only (n = 227) and among those women with GDM diagnosed by both the IADPSG’s criteria and the World Health Organization’s (WHO) criteria (n = 473) [11].

Sensitivity analysis was performed with re-inclusion of 130 SC women (3 lost to follow-up) and 112 UC women (3 lost to follow-up) entering the trial during Nov 2010 to July 2011 and further adjustment for other covariables.

## Results

### Characteristics of the trial women

The mean age of the 700 subjects in the trial was 29.8 (SD: 3.3) years and 95.1% of them were nulliparous and 97.0% were of Han ethnicity. These women had their first antenatal care visit at 10.6 (IQR: 9.3 to 12) gestational weeks and were enrolled into the trial at 26.3 (IQR: 25.4 to 27.3) gestational weeks. The 1-hour 50-gram GCT and 2-hour 75-gram OGTT results were similar between the two groups. Both groups had similar BMI at their first antenatal care visit and similar weight gain from their first antenatal care visit to the date when OGTT was performed. The women assigned to the SC group had a marginally shorter body height and had a marginally higher fasting PG at the OGTT than the UC group (Table 1).

**Table 1:** **Clinical and biochemical characteristics of pregnant women at or before enrollment by assignment**

	**Usual care (n = 361)**	**Shared care (n = 339)**	
**Variables**	**Mean (SD) or n(%)**	**Mean (SD) or n(%)**	**P value** ^**†**^
**At the 1** ^**st**^ **antenatal care visit**			
Age, year	29.7(3.2)	29.9(3.5)	0.475
Ethnicity			
Han	350(97.0%)	329(97.0%)	0.940
Others	11(3.1%)	10(3.0%)	
Smoking status			0.753
Ex-smoker	16(4.4%)	12(3.5%)	
Current smoker	3(0.8%)	4(1.2%)	
Alcohol intake			0.507
Ex-occasional drinker	97(26.9%)	79(23.3%)	
Current occasional drinker	14(3.9%)	16(4.7%)	
Body height, cm	163(4.5)	162(5.1)	0.091
Prepregnancy BMI, kg/m^2^	23.4(3.9)	22.9(3.6)	0.111
BMI classification			0.365
Below 18.5 (underweight)	23(6.4%)	25(7.4%)	
18.0-23.9 (normal weight)	202(56.0%)	206(60.8%)	
24.0-27.9 (overweight)	91(25.2%)	77(22.7%)	
28.0 and above (obesity)	45(12.5%)	31(9.1%)	
Parity			
0	342(95.0%)	323(95.3%)	0.870
1 and more	18(5.0%)	16(4.7%)	
Systolic BP, mmHg	107(10.2)	108(10.9)	0.742
Diastolic BP, mmHg	69(7.5)	70(7.8)	0.339
Gestational age at the first visit, weeks	10.8(2.3)	10.8(2.4)	0.593
**At screening for and diagnosis of GDM**			
GCT, mmol/L^‡^	8.9(8.3-9.8)	9.0(8.4-9.8)	0.364
Systolic BP, mmHg	109(10.1)	109(10.7)	0.786
Diastolic BP, mmHg	69(7.6)	70(7.5)	0.554
HbA1c, %	5.0(0.5)	5.0(0.5)	0.771
OGTT			
Fasting PG, mmol/L	5.0(0.5)	5.1(0.6)	0.084
1-h PG, mmol/L	10.0(1.3)	10.1(1.4)	0.366
2-PG, mmol/L	8.4(1.4)	8.4(1.2)	0.983
Fasting insulin, mIU/L^‡^	8.7(5.7-12.8)	9.0(5.6-12.8)	0.867
2-h insulin, mIU/L^‡^	86.1(51.1-128.8)	82.6(50.9-123.1)	0.703
Weight gain at OGTT, kg	9.7(4.4)	9.8(4.4)	0.774
Gestational age at OGTT, weeks^‡^	26.3(25.4-27.1)	26.3(25.4-27.4)	0.410

*Abbreviations:* *BMI* body mass index, *BP* blood pressure, *GDM* gestational diabetes mellitus, *GCT* glucose challenge test, *HbA1c* haemoglobin A1c, *OGTT* oral glucose tolerance test, *PG* plasma glucose.

^†^P values were derived from Chi-square Test, Fisher’s Exact Test, or Student T Test unless specified.

^‡^Data were reported as median (interquartile range) and P values were derived from Wilcoxon Two-Sample Test.

### Compliance of the SC women

The SC women had a moderate compliance to the intervention protocol with 66.7% attending all three individualised education sessions and 60.8% attending at least one group education session. There was a higher frequency reaching the physical activity target in the SC group than in the UC group. However, energy intake and HbA_1c_ were similar in the two groups. As a result, among the SC women who returned for 30^th^ and 34^th^ gestational week follow-ups, 93.5% and 80.6% achieved the glycaemic control target (Table 2).

**Table 2:** **Clinical and biochemical characteristics of pregnant women after enrollment to before delivery by assignment**

	**Usual Care (n = 361)**	**Shared Care (n = 339)**	
**Variables**	**Mean (SD) or n(%)**	**Mean (SD) or n(%)**	**P Value** ^**†**^
Attended individual education sessions			
One session	-	66(19.5%)	
Two sessions	-	47(13.9%)	
Three sessions	-	226(66.7%)	
Attended group education sessions			
None	-	133(39.2%)	
One session only	-	101(29.8%)	
Two and more sessions	-	105(31.0%)	
**At 30** ^**th**^ **gestational weeks**			
Attended the 30 gestational week follow-up	-	247(72.9%)	
Weight gain from entry to 30^th^ gestational week, kg/week^a^		0.1(0.5)	
Number of self-monitoring of blood glucose	-	52(22)	
Glycaemic control target achieved	-	231(93.5%)	
Energy intake, Kcal/day	-	1832(347)	
**At 34** ^**th**^ **gestational weeks**			
**Attended the 34** ^**th**^ **gestational week follow-up**	221(61.2%)	252(74.3%)	<0.001
Weight gain from entry to 34^th^ gestational week, kg/week^a^	0.31(0.32)	0.23(0.35)	0.012
Total number of self-monitoring of blood glucose	-	72(24)	
Glycaemic control target achieved	-	203(80.6%)	
Energy intake, Kcal/day^aa^	1899(352)	1886(344)	0.718
HbA1c, %^ab^	5.2(0.5)	5.2(0.5)	0.252
HbA1c, mmol/mol	33(3)	33(3)	0.252
Insulin use	1(0.3%)	4(1.2%)	0.156
Commuting in the past month^ac^			0.731
Stay at home	103(46.6%)	114(45.2%)	
Public transportation	64(29.0%)	71(28.3%)	
Driving	39(17.7%)	53(21.1%)	
Walking/biking	15(6.8%)	13(5.2%)	
Engaged in leisure time physical activity in the past month: yes vs. no^ac^	172(77.8%)	212(84.5%)	0.065
Reaching physical activity target in the past month (30 min per time, ≥7 times per week)^ac^	57(25.8%)	86(34.3%)	0.045
Patient health questionnaire (PHQ-9) Score as a continuous variable^a^	5.1(2.3)	4.9(2.6)	0.389
Patient health questionnaire (PHQ-9) Score as a categorical variable^a^			0.548
≥15 (major depression)	1(0.5%)	2(0.8%)	
10-14 (minor depression)	8(3.5%)	14(5.6%)	
<10	211(95.9%)	236(93.7%)	
**At delivery**			
Weight gain during pregnancy, kg^b^	15.7(6.4)	15.5(6.5)	0.785
Weight gain from 34^th^ gestational week to delivery, kg/week^c^	0.62(0.54)	0.61(0.60)	0.896
Insulin use	18(5.0%)	25(7.4%)	0.188
The number of obstetric visits since 28^th^ gestational weeks^d^	7.6(2.5)	7.8(3.0)	0.509

^†^P values were derived from Chi-square Test, Fisher’s Exact Test, or Student T Test unless specified.

Valid sample size of the usual care vs. the shared care: ^a^220 vs. 252, ^aa^136 vs. 252, ^ab^220 vs. 250, ^ac^221 vs. 251, ^b^322 vs. 305; ^c^203 vs. 231; ^d^164 vs. 207.

### Pregnancy outcomes

The rate of macrosomia was 11.2% (38/339) in the SC group compared to 17.5% (63/361) in the UC group with RR of 0.64 (95% CI 0.44-0.93) (Table 3). The rate of PIH was 8.0% (27/339) in the SC group compared to 4.4% (16/361) in the UC group with RR of 1.80 (0.99-3.28). After adjustment for borderline significant variables, i.e., body height and fasting PG at OGTT, the RRs of SC versus UC for macrosomia and PIH remained the same (macrosomia:0.66, 95% CI: 0.45-0.95; PIH: 1.77, 95% CI: 0.98-3.21).

**Table 3:** **Pregnancy outcomes of women with gestational diabetes mellitus by assignment**

	**UC (n = 361)**	**SC (n = 339)**		**SC vs. UC**	**SC vs. UC**
**Outcomes**	**Mean (SD) or n(%)**	**Mean (SD) or n(%)**	**P value**	**Unadjusted RR (95% CI)**	**Adjusted RR** ^**†**^ **(95% CI)**
Delivery at a tertiary hospital	237(65.7%)	237(69.9%)	0.228		
Gestational weeks at delivery	39.2(2.1)	39.4(2.9)	0.243		
**Primary outcome**					
Birth weight ≥4.0 kg	63(17.5%)	38(11.2%)	0.019	0.64(0.44-0.93)	0.66(0.45-0.95)
**Secondary outcome**					
Pregnancy-induced hypertension	16(4.4%)	27(8.0%)	0.052	1.80(0.99-3.28)	1.77(0.98-3.21)
**Other infant outcomes**					
Birth weight, g	3469(574)	3371(530)	0.021		
Body stature of neonates, cm	50.2(1.9)	50.1(1.8)	0.248		
Infant male gender	190(52.9%)	204(60.4%)	0.048		
Large for gestational age^‡^	72(19.9%)	44(13.0%)	0.013	0.65(0.46-0.92)	0.66(0.47-0.93)
Birth weight ≥4.5 kg	10(2.8%)	7(2.1%)	0.541	0.75(0.29-1.94)	0.75(0.28-1.99)
Birth weight < 2.5 kg	14(3.9%)	14(4.1%)	0.865	1.06(0.52-2.20)	1.00(0.48-2.08)
Apgar score at 1 min <7	7(1.9%)	0(0%)	0.016		
Neonatal hypoglycemia^§^	4(1.1%)	2(0.6%)	0.457	0.53(0.10-2.89)	0.55(0.10-3.09)
Birth trauma or shoulder dystocia	0	0	-	-	-
Bone fracture	0	0	-	-	-
Stillbirth or neonatal death	4(1.1%)	4(1.2%)	0.929	1.06(0.27-4.22)	1.01(0.27-3.84)
**Other maternal or delivery outcomes**					
Induced labor	1(0.3%)	0	-		
Preterm birth	28(7.8%)	18(5.3%)	0.192	0.68(0.39-1.21)	0.64(0.36-1.14)
Premature rupture of membrane	69(19.1%)	49(14.5%)	0.100	0.76(0.54-1.06)	0.74(0.53-1.04)
Caesarean delivery	233(64.5%)	239(70.5%)	0.093	1.09(0.99-1.21)	1.09(0.98-1.21)
Preeclampsia	8(2.2%)	18(5.3%)	0.031	2.40(1.06-5.44)	2.35(1.05-5.26)
Gestational hypertension	8(2.2%)	9(2.7%)	0.706	1.20(0.47-3.07)	1.18(0.46-3.05)

*Abbreviations:* *SC* shared intensive care, *UC* usual care.

^†^Adjusted RR, derived from prevalence ratios that were adjusted for fasting plasma glucose and maternal body height that were marginally significant.

^‡^Large for gestational age was defined by gender and gestational age specific 90^th^ percentiles.

^§^Defined as capillary blood glucose <1.7 mmol/L.

Infants born to the women in the SC group had a lower birth weight by 98 grams (SC vs. UC: 3469 versus 3371 grams, P = 0.021). The rate of LGA was 13.0% (44/339) in the SC group and 19.9% (72/361) in the UC group with a RR of 0.65 (95% CI 0.46-0.92). After adjustment for borderline significant variables, the RR of SC versus UC for LGA was 0.66 (95% CI: 0.47-0.93). The rate of preeclampsia was significantly higher in the SC group than in the UC group (5.3% vs. 2.2%, P = 0.031) with unadjusted RR of 2.40 (95% CI: 1.06-5.44) and adjusted RR of 2.35 (95% CI: 1.05-5.26). Gestational hypertension was similar in the two groups. The rate of Apgar score <7 was significantly reduced from 1.9% (7/361) in the UC to nil in the SC (P = 0.016). However, the rates of hypoglycemia in both groups were low and not statistically significant between the two groups. Over 60% of women had caesarean delivery which was marginally higher in the SC group than in the UC group with adjusted RR of 1.09 (95% CI: 0.99-1.21). Low birth weight defined as birth weight <2500 grams, stillbirth and neonatal death, preterm birth and premature rupture of membrane were all similar in the two groups (Table 3).

### Subgroup analysis

The prevalence of overweight and obesity was 23.4% and 9.7% in the GDM women diagnosed by the IADPSG’s criteria only and 24.3%11.4% in the GDM women diagnosed by both the IADPSG’s and the WHO’s (P = 0.723). The SC reduced birth weight of 68 grams among women with GDM diagnosed by the IADPSG’s criteria only and 111 grams among women with GDM diagnosed by both the IADPSG’s criteria and the WHO’s criteria (Table 4). The RRs for macrosomia were similar among women with GDM diagnosed by the IADPSG’s and by both sets of criteria but LGA was only significant among women with GDM diagnosed by both the IADPSG’s criteria and the WHO’s criteria but not diagnosed by the IADPSG’s criteria only. The rates of PIH were not statistically significant between the SC group and the UC group with diagnosed GDM either by the IADPSG’s only or by both sets of criteria. The magnitude of the RR for PIH was much larger numerically among women with GDM diagnosed by IADPSG’s only than by both sets of diagnostic criteria.

**Table 4:** **Subgroup analysis of predefined pregnancy outcomes of women with gestational diabetes mellitus by assignment**

	**UC**	**SC**		**SC vs. UC**	**SC vs. UC**
**Outcomes**	**Mean (SD) or n(%)**	**Mean (SD) or n(%)**	**P value**	**Unadjusted RR (95% CI)**	**Adjusted RR** ^**†**^ **(95% CI)**
**Among GDM diagnosed by the IADPSG’s criteria only**					
n	117	110			
Birth weight, g	3522(590)	3454(496)	0.352		
Birth weight ≥4.0 kg	25(21.4%)	14(12.7%)	0.085	0.60(0.32-1.09)	0.62(0.34-1.13)
Large for gestational age^‡^	27(23.1%)	21(19.1%)	0.462	0.83(0.50-1.37)	0.88(0.54-1.46)
Pregnancy-induced hypertension	3(2.6%)	7(6.4%)	0.163	2.48(0.66-9.36)	2.48(0.67-9.26)
**Among GDM diagnosed by both IADPSG’s and WHO’s criteria**					
n	244	229			
Birth weight, g	3443(565)	3332(542)	0.030		
Birth weight ≥4.0 kg	38(15.6%)	24(10.5%)	0.097	0.67(0.42-1.08)	0.67(0.41-1.09)
Large for gestational age^‡^	45(18.4%)	23(10.0%)	0.009	0.54(0.34-0.87)	0.52(0.33-0.84)
Pregnancy-induced hypertension	13(5.3%)	20(8.7%)	0.146	1.64(0.84-3.22)	1.56(0.79-3.06)

*Abbreviations:* *SC* shared intensive care, *UC* usual care, *IADPSG* International Association of Diabetes and Pregnancy Study Group, *WHO* World Health Organization.

^†^Adjusted RR, derived from prevalence ratios that were adjusted for fasting plasma glucose and maternal body height that were marginally significant.

^‡^Large for gestational age was defined by gender and gestational age specific 90^th^ percentiles.

### Sensitivity analysis

In the sensitivity analysis, all the variables listed in Table 1 were non-significant between the SC group and the UC group, including body height (P = 0.869) and fasting glucose on OGTT (P = 0.342). Inclusion of those women who entered the trial during Nov 2010 to July 2011 attenuated the intervention effect on macrosomia and PIH to non-significance (Table 5). However, LGA (SC vs. UC: 18.9% vs. 14.0%, P = 0.040) and Apgar score at 1 min <7 (SC vs. UC: 0.6% vs. 3.0%, P = 0.008) remained significant. Preterm birth was also less frequent in the SC group than in the UC group (4.7% vs. 7.9%, P = 0.047). On the other hand, caesarean delivery was more frequent in the SC group than in the UC group (71.5% vs. 64.3%, P = 0.018).

**Table 5:** **Sensitivity analysis of pregnancy outcomes of 936 women with gestational diabetes mellitus by assignment**

	**UC (n = 470)**	**SC (n = 466)**		**SC vs. UC**
**Outcomes**	**Mean (SD) or n(%)**	**Mean (SD) or n(%)**	**P value**	**Unadjusted RR (95% CI)**
Delivery at a tertiary hospital	314(66.8%)	334(71.7%)	0.107	
Gestational weeks at delivery	39.1(2.1)	39.4(2.5)	0.063	
**Primary outcome**				
Birth weight ≥4.0 kg	73(15.6%)	58(12.5%)	0.170	0.80(0.58-1.10)
**Secondary outcome**				
Pregnancy-induced hypertension	22(4.7%)	33(7.1%)	0.118	1.51(0.90-2.56)
**Other infant outcomes**				
Birth weight, g	3445(587)	3415(527)	0.412	
Body stature of neonates, cm	50.2(2.0)	50.2(1.8)	0.694	
Infant male gender	244(51.9%)	271(58.2%)	0.055	
Large for gestational age^†^	89(18.9%)	65(14.0%)	0.040	0.74(0.55-0.99)
Birth weight ≥4.5 kg	12(2.6%)	14(3.0%)	0.679	1.17(0.55-2.51)
Birth weight < 2.5 kg	22(4.7%)	15(3.2%)	0.251	0.68(0.36-1.31)
Apgar score at 1 min <7	14(3.0%)	3(0.6%)	0.008	0.22(0.06-0.75)
Neonatal hypoglycemia^‡^	4(0.9%)	2(0.4%)	0.418	0.50(0.09-2.74)
Birth trauma or shoulder dystocia	0	0	-	-
Bone fracture	0	0	-	-
Stillbirth or neonatal death	5(1.1%)	5(1.1%)	0.989	1.01(0.29-3.46)
**Other maternal or delivery outcomes**				
Induced labor	2(0.4%)	0	0.500	
Preterm birth	37(7.9%)	22(4.7%)	0.047	0.60(0.36-1.00)
Premature rupture of membrane	83(17.7%)	74(15.9%)	0.466	0.90(0.68-1.20)
Caesarean delivery	302(64.3%)	333(71.5%)	0.018	1.11(1.02-1.22)
Preeclampsia	12(2.6%)	21(4.5%)	0.105	1.76(0.88-3.55)
Gestational hypertension	10(2.1%)	12(2.6%)	0.651	1.21(0.53-2.77)

*Abbreviations:* *SC* shared intensive care, *UC* usual care.

^†^Large for gestational age was defined by gender and gestational age specific 90^th^ percentiles.

^‡^Defined as capillary blood glucose <1.7 mmol/L.

After further adjusting for whether the glycaemic control target had been achieved at the 30^th^ and 34^th^ gestational weeks in the main analysis, the magnitude of the RRs for macrosomia (RR: 0.74, 95% CI: 0.42-1.30) and for LGA (RR: 0.78, 95% CI: 0.47-1.29) were attenuated. However, the RR for PIH was almost unchanged (RR: 1.78, 95% CI: 0.79 to 3.98) and that for preeclampsia was even slightly increased (RR: 2.43, 95% CI: 0.86-6.86).

Further explorations of the role of physical activity in the increased risks of PIH and preeclampsia were made by including whether reaching the physical activity target as a covariable. Adjustment for whether achieving the physical activity target increased the RRs of SC vs. UC for PIH (RR:2.52, 95% CI: 1.10-5.79) and preeclampsia (RR:3.65, 95% CI: 1.06-12.58).

## Discussion

Gestational diabetes is associated with adverse pregnancy outcomes and intensive intervention to normalise blood glucose has been shown to improve outcomes among women with mild GDM diagnosed by the WHO’s criteria [11] and the Fourth International Workshop-Conference on GDM’s criteria [9,10]. Using a pragmatic randomised trial design, we demonstrated that intensive lifestyle intervention was able to improve pregnancy outcomes among women diagnosed by the new IADPSG’s criteria [13].

In the ACHOIS [10], intensive management reduced the rate of serious adverse pregnancy perinatal complications but the US trial did not find that intensive management reduced the rate of infant morbidity [9]. In our study, none of the pregnancies had birth trauma, shoulder dystocia, bone fracture and nerve palsy, possibly due to the high rate of caesarean delivery of 60-70%, a rate much higher than in US and Australia [9,10]. There was around 1% of stillbirth or neonatal death in both groups which was comparable to that in the US trial [9]. In the ACHOIS, there was a 53% risk reduction in macrosomia and 38% risk reduction in LGA [10] while the respective rates in the US trial were 59% and 51% [9]. Thus, the risk reductions of 34-36% for macrosomia and LGA in our trial were smaller than those in the ACHOIS and the US trial, which may be partially attributable to the nature of our pragmatic trial.

In contrast to the two trials conducted in US and Australia that showed decreased risk of preeclampsia or PIH with intervention [9,10], our study did not find that the intervention was able to reduce the risk of PIH but suggested a significant increased risk of preeclampsia. The ACHOIS [10] and the US trial [9] did not document physical activity. Two small trials [28] failed to generate conclusive findings due to shortfall of power (RR of moderate physical activity for preeclampsia:0.31, 95% CI: 0.01 to 7.09). In our study, adjustment for physical activity increased but not decreased the RRs for PIH and preeclampsia, suggesting that physical activity was unlikely to account for the increased risks of PIH and preeclampsia in the SC group.

Although being supported by strong epidemiological evidence from the HAPO study, there are still ongoing debates on use of the new IADPSG’s criteria [29]. One of the concerns is that there is no clinical evidence showing the benefits of intervention for the fasting PG threshold in the new criteria [30]. There are also ongoing debates whether prepregnancy obesity/overweight or IADPSG-defined GDM contributes more to LGA [31,32]. In addition to overall effectiveness for IADPSG’s criteria-defined GDM, our trial also showed that the effect size of the intervention for macrosomia was similar in GDM diagnosed by the IADPSG’s only and by both the WHO’s and the IADPSG’s although the prevalence of obesity and overweight was similar in the two groups, supporting use of the IADPSG’s criteria in Chinese pregnant women. However, the intervention for LGA seems to be more effective for WHO-defined GDM than IADPSG-defined GDM, which needs further elucidation. In the subgroup analysis, the RR of the intervention for PIH was numerically larger among women with GDM diagnosed by the IADPSG’s criteria only than by both sets of criteria. However, it is worth noting that the sample size of PIH cases in the IADPSG’s criteria only group was small and we can not rule out that the larger RRs in the former group was due to chance.

Our study has strong pragmatic implications, especially for developing countries. In China, the prevalence of diabetes [33] and GDM [17] has been increasing rapidly. Capacity building and strengthening of the care system are in high demand for coping with the marked increase in the prevalence of diabetes and GDM. Shared management between different care teams is one way to improve the quality of care. In this randomised study, shared care between TWCHC GDM clinic and hospitals within a 3-tier system with particular focus on lifestyle modifications reduced risks of macrosomia and LGA by 34-36% in Chinese women with mild GDM that were, at least, partially attributable to achievement of the glycaemic control target.

This trial had strengths and limitations. First, this was a population-based pragmatic quality improvement program implemented within a 3-tier antenatal care system in a developing country. In this regard, experts have now recommended care professionals to use pragmatic and multipronged strategies to translate evidence to clinical practice with ongoing evaluation [34]. Second, only two-thirds of women were evaluated for clinical assessment at the 34^th^ gestational week follow-up. The results of the analysis of these intermediate measurements need to be interpreted with caution. Third, although women recruited in the trial were blinded to their assignment status and the two groups of women were asked to come back on follow-up visits at different times and different areas, the care providers were not blinded to the status of assignment. The increased awareness of GDM in both the subjects and care providers might change the practice and bias due to the increased awareness could not be excluded. Nevertheless, the similar rates of delivery at a tertiary care hospital and use of insulin at delivery in the two arms suggested that the main effects were unlikely to be severely biased.

## Conclusion

In this randomised quality improvement program, lifestyle intervention in a 3-tier’s shared management system reduced rates of macrosomia, LGA and neonatal Apgar score at 1 min <7 in women with mild GDM diagnosed with the IADPSG’s criteria. Our findings may be applicable to developing countries where antenatal care is shared among different care providers. However, the increased risk of preeclampsia needs further investigations.

## Additional file

Additional file 1:
**Flow Chart Showing the Care Protocols for Women with GDM and Evaluation of Pregnancy Outcomes.**

## Abbreviations

ACHOIS: Australian carbohydrate intolerance study in pregnant women
BMI: Body mass index
CI: Confidence interval
GCT: Glucose challenge test
GDM: gestational diabetes mellitus
IADPSG: International association of diabetes and pregnancy study groups
IQR: Interquartile range
LGA: Large for gestational age
OGTT: Oral glucose tolerance test
PG: Plasma glucose
PHQ: Patient health questionnaire
RR: Relative risk
SC: Shared care
SD: Standard deviation
SMBG: Self monitoring of blood glucose
TWCHC: Tianjin Women and Children’s Health Centre
UC: Usual care
WCHC: Women and Children’s Health Centres
WHO: World Health Organization
