# Erratum to: a randomised translational trial of lifestyle intervention using a 3-tier shared care approach on pregnancy outcomes in Chinese women with gestational diabetes mellitus but without diabetes

**DOI:** 10.1186/s12967-014-0332-9

**Published:** 2015-02-21

**Authors:** Xilin Yang, Huiguang Tian, Fuxia Zhang, Cuiping Zhang, Yi Li, Junhong Leng, Leishen Wang, Gongshu Liu, Ling Dong, Zhijie Yu, Gang Hu, Juliana CN Chan

**Affiliations:** Department of Epidemiology and Biostatistics, School of Public Health, Tianjin Medical University, Tianjin, 300070 China; Department of Medicine and Therapeutics, Hong Kong Institute of Diabetes and Obesity, The Chinese University of Hong Kong and The Chinese University of Hong Kong-Prince of Wales Hospital-International Diabetes Federation Centre of Education, Hong Kong, China; Tianjin Women and Children’s Health Centre, Tianjin, China; Population Cancer Research Program and Department of Pediatrics, Dalhousie University, Halifax, Canada; Chronic Disease Epidemiology Laboratory, Pennington Biomedical Research Center, Baton Rouge, Louisiana USA

## Erratum

After the publication we noticed the misspelling of author Gongshu Liu’s name as Gongsu Liu in our article [1]. We apologise for this.
