# Supplementary material for: A randomised translational trial of lifestyle intervention using a 3-tier shared care approach on pregnancy outcomes in Chinese women with gestational diabetes mellitus but without diabetes
Source: J Transl Med. 2014 Oct 28;12:290. doi: 10.1186/s12967-014-0290-2 (PMC4213554; doi:10.1186/s12967-014-0290-2)
Supplement: Additional file 1: — Flow Chart Showing the Care Protocols for Women with GDM and Evaluation of Pregnancy Outcomes. [file 12967_2014_290_MOESM1_ESM.doc]

Additional file 1. Flow Chart Showing the Care Protocols for Women with GDM and Evaluation of Pregnancy Outcomes

| **Gestational weeks** | <24 | 24 | 25 | 26 | 27 | 28 | 29 | 30 | 31 | 32 | 33 | 34 | 35 | 36 | >36 |
| --- | --- | --- | --- | --- | --- | --- | --- | --- | --- | --- | --- | --- | --- | --- | --- |
| Registration at a primary care hospital <12 gestational week |  |  |  |  |  |  |  |  |  |  |  |  |  |  |  |
| Questionnaires |  |  |  |  |  |  |  |  |  |  |  |  |  |  |  |
| BW /BP/lifestyle |  |  |  |  |  |  |  |  |  |  |  |  |  |  |  |
| **Primary care hospital** | | | | | | | | | | | | | | | |
| Antenatal visits to obstetricians | Every 4 weeks | | | | | Every 2 weeks | | | | |  |  |  |  |  |
| Mother: BW/BP/urine protein | √ | | | | | √ | | | | |  |  |  |  |  |
| Fetus: IUG/HR/position | √ | | | | | √ | | | | |  |  |  |  |  |
| Screening for Down’s | √ |  |  |  |  |  |  |  |  |  |  |  |  |  |  |
| Screening for GDM |  | √ | | | | |  |  |  |  |  |  |  |  |  |
| Questionnaires |  | √ | | | | |  |  |  |  |  |  |  |  |  |
| BW /BP/lifestyle |  | √ | | | | |  |  |  |  |  |  |  |  |  |
| **Secondary/tertiary care hospital** | | | | | | | | | | | | | | | |
| Antenatal visit to obstetricians |  |  |  |  |  |  |  |  |  |  | Every 2 weeks | | | Every week | |
| Mother: BW/BP/urine protein |  |  |  |  |  |  |  |  |  |  | √ | | | √ | |
| Fetus: IUG/HR/position |  |  |  |  |  |  |  |  |  |  | √ | | | √ | |
| Delivery by obstetricians |  |  |  |  |  |  |  |  |  |  |  |  |  |  | √* |
| **For women with GCT plasma glucose ≥ 7.8 mmol/L** | | | | | | | | | | | | | | | |
| **TWCHC** |  |  |  |  |  |  |  |  |  |  |  |  |  |  |  |
| 75-g 2-h OGTT |  | Confirming GDM & assessing eligibility | | | | |  |  |  |  |  |  |  |  |  |
| **Usual care group (antenatal care as above)** | | | | | | | | | | | | | | | |
| **TWCHC** |  |  |  |  |  |  |  |  |  |  |  |  |  |  |  |
| Group education |  | One session upon diagnosis of GDM | | | | |  |  |  |  |  |  |  |  |  |
| Review of hyperglycemia |  | One week after diagnosis of GDM | | | | |  |  |  |  |  |  |  |  |  |
| **Shared care group (antenatal care as above)** | | | | | | | | | | | | | | | |
| **TWCHC** |  |  |  |  |  |  |  |  |  |  |  |  |  |  |  |
| Group education |  | One session upon diagnosis of GDM | | | | |  |  |  |  |  |  |  |  |  |
| Review of hyperglycemia |  | One week after diagnosis of GDM | | | | |  |  |  |  |  |  |  |  |  |
| Individual counseling |  | One session upon diagnosis of GDM | | | | |  | √ |  |  |  | √ |  |  |  |
| Diet, physical activity, SMBG & insulin if indicated |  | √ | | | | | √ | √ | √ | √ | √ | √ | √ | √ | √ |
| Group education at TWCHC or a place close to their neighborhood |  |  |  |  | √ |  | √ |  |  |  | √ |  |  |  |  |

GDM, gestational diabetes mellitus; GCT, 1-h 50-g glucose challenge test; BW, body weight; BP, blood pressure; IUG, intrauterine growth; HR, heart rate; TWCHC, Tianjin Women and Children’s Health Centre; SMBG, self monitoring of blood glucose.

*Outcomes: Pregnancy induced hypertension (PIH) for mothers and macrosomia for infants.
